# Alterations in static and dynamic topological properties of brain functional network after chronic high altitude exposure: a panel study

**DOI:** 10.3389/fnagi.2026.1773258

**Published:** 2026-07-27

**Authors:** Siyao Zeng, Yang Zhou, Sijia Guo, Yang Ou, Tingting Yang, Chong Xue, Xinqin Liu, Jie Liu, Jianbin Zhang, Wenjing Luo, Xiaoming Chen

**Affiliations:** 1 The Ministry of Education Key Laboratory of Hazard Assessment and Control in Special Operational Environments, Shaanxi Provincial Key Laboratory of Environmental Health Hazard Assessment and Protection, Shaanxi Provincial Key Laboratory of Free Radical Biology and Medicine, Department of Occupational and Environmental Health, School of Public Health, Fourth Military Medical University, Xi’an, China; 2 The General Hospital of PLA Xizang Military Area Command, Lhasa, China

**Keywords:** cognitive impairment, dynamic global network property, graph theoretic analysis, high altitude, magnetic resonance imaging

## Abstract

**Background:**

Long-term exposure to a high-altitude (HA) hypoxic environment induces cognitive impairments, but the underlying mechanisms remain unclear. Notably, the specific topological features of brain networks in this context have not been thoroughly explored. This study investigated graph-based topological characteristics of brain networks from static and dynamic perspectives, and examined how these alterations contribute to the chronic HA-induced cognitive changes.

**Methods:**

This longitudinal panel study enrolled 49 college freshmen who relocated from low altitude to a HA region in Tibet. Comprehensive cognitive assessments and brain magnetic resonance imaging (MRI) were performed at baseline, 2 years, and 4 years. Resting-state functional MRI data were used to characterize the topological organization of functional brain networks, encompassing both nodal and global network properties.

**Results:**

Behavioral assessments showed persistent impairments in cognitive functions following prolonged HA exposure. Neuroimaging analyses consistently identified the left superior parietal gyrus, left inferior parietal lobule, and right Heschl’s gyrus as key nodes for both static and dynamic nodal properties. After 4 years of HA exposure, all dynamic global network metrics were elevated relative to baseline values, and these alterations were associated significantly with cognitive changes. Specifically, increased variances of characteristic path length (Lp) and global efficiency (Eglob) correlated with working memory decline.

**Conclusion:**

Our findings demonstrate that variances in Lp and Eglob contribute significantly to chronic hypoxia-induced cognitive impairment. Collectively, these results provide novel evidence that dynamic functional instability, as reflected by altered fluctuations in Lp and Eglob, plays a pivotal role in driving hypoxia-related cognitive decline.

## Introduction

Because high-altitude (HA) environments have a reduced atmospheric oxygen partial pressure compared with low-altitude locations, continued exposure to HA conditions poses a unique physiological challenge to the human central nervous system. Accumulating evidence from recent empirical studies indicates that prolonged or acute HA exposure induces significant alterations in brain functional networks, which in turn contribute to observable behavioral deficits, such as impairments in attention, memory, and motor coordination (Chen et al., 2021; Zhou et al., 2024). These findings highlight the critical need for rigorous analytical frameworks to quantify and interpret the topological reorganization of brain networks under the hypoxic conditions of HA environments.

Brain functional connectivity can be non-invasively quantified via resting-state fMRI (rs-fMRI) based on BOLD signal fluctuations coupled to spontaneous neuronal activity, and aberrant connectivity has been widely reported across multiple cognitive disease cohorts (Deogaonkar et al., 2016; Zhang et al., 2013; Lv et al., 2018). Advances in graph theory have made it a mainstream analytical tool for quantifying the topological properties of brain functional networks, including small-world attributes, modular organization, and hub-node distribution (Bullmore and Sporns, 2009), and it is commonly used to map network reorganization in healthy and clinical populations (Chen et al., 2024; Eryilmaz et al., 2022; Wang et al., 2024). For example, using graph theory, Eryilmaz et al. (2022) demonstrated that node dysfunctions in prefrontal and parietal regions are associated closely with cognitive impairments in schizophrenia (SZ) patients, underscoring the method’s value for linking network topology to functional outcomes. In the context of HA exposure, prior research has begun to apply static graph theory to explore network reorganization. Xin et al. (2020) reported that after 2 years of HA residence, immigrants exhibited significant changes from baseline in degree centrality and nodal efficiency across multiple brain regions, including those involved in attention control, reaction, memory, and motor coordination. These findings suggest that HA exposure may disrupt the optimal balance between neural integration (inter-regional communication) and segregation (local specialization).

However, static graph theory is inherently limited by its focus on “average” network properties derived from entire rs-fMRI scanning sessions. This limitation precludes the capture of time-varying fluctuations in functional connectivity, which are increasingly recognized as essential for understanding context-specific brain states (e.g., task engagement, pathological progression) and their relationship to network structure. Dynamic graph theory can address this gap by enabling analysis of how network topology evolves over time through the application of time-varying analytical techniques (Li L. et al., 2024). For example, Wang et al. (2024) employed dynamic graph theory to investigate topological changes in brain networks among patients with retinal detachment (RD) and identified significant alterations in both global network properties and node-specific characteristics. Importantly, these dynamic changes were found to correlate with clinical parameters, revealing mechanistic relationships that would not have been revealed by static models.

Despite technical advances in the application of dynamic network analysis in neuroscience, existing neuroimaging studies exploring HA hypoxia-induced brain functional remodeling have predominantly adopted cross-sectional designs. Cross-sectional comparisons have notable methodological constraints, as they do not permit separation of inherent inter-individual brain discrepancies from hypoxia-driven adaptive neural remodeling and cannot trace the progressive trajectory of cerebral plasticity as well as concomitant cognitive transformations after chronic HA exposure (Li and Wang, 2022; Ren et al., 2025). Against this backdrop, the present study employed a rigorous 4-year longitudinal panel design combined with dynamic graph theoretical analysis, which fundamentally addresses the dual shortcomings of conventional cross-sectional sampling and static network analytical strategies. This unique framework enables continuous tracking of time-varying topological reorganization in whole-brain functional networks throughout HA adaptation, captures transient network fluctuations masked by traditional average-based static analyses, and further establishes explicit temporal associations between progressive neural network remodeling and longitudinal cognitive changes. These novel, dynamic mechanistic insights substantially advance the knowledge gained from prior HA neuroimaging findings based solely on cross-sectional static observations.

To date, systematic investigations of time-resolved dynamic alterations in whole-brain functional networks during chronic HA exposure remain scarce, leaving a critical knowledge gap regarding the spatiotemporal mechanisms of hypoxia-induced brain and cognitive adaptation. Static graph analyses can only reflect averaged network topological properties, failing to capture transient, time-dependent functional changes that may underlie progressive neural adaptive responses or subtle cognitive fluctuations under hypoxic conditions. Building upon our prior work (Zeng et al., 2026), in which we performed functional connectivity calculations focused only on pairwise correlation strengths between brain regions, the current study extends beyond simple FC correlation values to systematic graph-theoretical topological quantification. To fill this research gap, the present study included a 4-year longitudinal follow-up of healthy young immigrants exposed to the HA environment, focusing on longitudinal alterations in brain functional networks and corresponding cognitive performance. By integrating complementary static and dynamic graph theoretical approaches, this study comprehensively characterizes both stable static features and time-varying spatiotemporal patterns of HA-related brain network reorganization, aiming to deepen the systematic understanding of how chronic HA exposure reshapes the hierarchical organization and dynamic functional flexibility of the human brain.

## Materials and methods

### Study design

The experimental group for this study consisted of students from the Shaanxi–Tibet immigrant cohort who were admitted to Tibet University in Lhasa, China, which has a mean altitude of 3,658 m, for a higher education term of 4 years. The participants completed a series of questionnaires collecting data regarding their medical history, history of exposure to HA environments, academic performance, and sociodemographic characteristics, including the education, vocation, and socioeconomic status of the students’ parents. Data collection began in July 2014, and follow-up investigations including neuropsychological assessments and MRI scan were performed in May 2016 and May 2018. During the follow-up assessments, participants were asked to provide information about the length of time they had spent at HA, any mountain climbing experience, any visits to low-altitude regions, and any change in medical history that had occurred during the study period. The protocol for this study was approved by the Ethics Committee of the Medical Faculty of Fourth Military Medical University (registry no. KY20143344-1), and the study adhered to the ethical principles for medical research as stated in the Declaration of Helsinki.

The control group consisted of healthy college students for whom longitudinal MRI data were available for download from the open access Southwest University Longitudinal Imaging Multimodal (SLIM) Brain Data Repository. This repository contains long-term test-retest multimodal brain imaging data acquired from healthy young Chinese adults, all of whom were university freshmen or sophomores with fluent Chinese proficiency. All participants were recruited in Southwest China, and scans were acquired using a 3.0-T Siemens Trio MRI system and standardized protocols. We selected control participants to match the experimental group for age and gender. Consistent demographic profiles, a rigorous longitudinal design, and stable imaging parameters make this database highly comparable to the experimental cohort. Each participant in the SLIM cohort completed three longitudinal scanning sessions. The use of SLIM data in the present study was approved by the Research Ethics Committee of the Brain Imaging Centre of Southwest University.

### Participants

Participants in the experimental group were recruited from healthy high school graduates of the Shaanxi–Tibet immigrant cohort. In July 2014, 120 high school graduates from Shaanxi Province were admitted to Tibet University (Lhasa, China; average altitude: 3,658 m) to pursue a 4-year undergraduate program. All admitted graduates and their parents were contacted via telephone and mail to invite their participation in this study. Of the 120 initially invited individuals, 70 consented to participate (total response rate, 58.3%). Participants were enrolled in the study if they met the following inclusion criteria: (1) age 17–20 years; (2) altitude of permanent residence is < 900 m; (3) no high-altitude exposure ( ≥ 2,500 m) in the past; (4) no history of smoking, alcohol, and drug abuse; (5) no history of medications that may affect cognitive function or carbonic anhydrase activity; and (6) no chronic or genetic diseases. All enrolled participants in this study signed the informed consent form prior to the experiment on the premise of fully understanding the content of the experiment. One participant was excluded due to a documented history of prior HA exposure, resulting in a final sample size of 69 for subsequent analyses.

Baseline investigations, including neuropsychological assessments and MRI scans, were conducted in July 2014 in Xi’an, China (altitude: 466 m). Follow-up investigations, neuropsychological assessments and MRI scans were carried out in May 2016 and May 2018 in Lhasa. Both at baseline and during each follow-up investigation, physical examinations were performed to evaluate participants’ overall health status. At baseline, 49 of 69 participants completed MRI and neuropsychological testing, while 20 did not undergo scanning. The cohort was followed at 2 and 4 years, with 62 participants retained at both follow-ups. All 49 baseline MRI participants remained in the study and completed the follow-up scans, with no attrition in this group. Of the 20 non-MRI participants, 3 were lost by the 2-year follow-up, leaving 17 who did not participate in subsequent MRI assessments. The final analytic sample with complete longitudinal MRI data consisted of 49 participants.

Control participants were screened from the SLIM Brain Data Repository for eligibility based on an age of 17–20 years, status as a university freshman or sophomore, and fluency in Chinese. Control participants were excluded if they met any one of the following exclusion criteria: (1) contraindication to MRI, such as claustrophobia, presence of a metal implant, Meniere’s syndrome, or a history of fainting within the previous 6 months; (2) current diagnosis of a psychiatric or neurological disorder; (4) psychiatric drug use within the previous 3 months; (5) pregnancy; or (6) a history of head trauma. MRI data were collected from three time points.

### Neuropsychological tests

All study participants completed in-depth neuropsychological assessments that evaluated a wide range of cognitive functions through collection of the following data: (1) visual memory data, including immediate visual memory (IVIM) and delayed visual memory (DVIM) as indicators of working memory of figures and shapes; (2) verbal memory data, including immediate verbal memory (IVBM) and delayed verbal memory (DVBM) as measures of working memory of words; (3) simple reaction time data, including visual simple reaction time (VSRT) and auditory simple reaction time (ASRT) as indicators of psychomotor function in response to a simple stimulus; (4) recognition reaction time data, including visual recognition reaction time (VCRT) and auditory recognition reaction time (ACRT) as measures of psychomotor function in response to target/distractive stimuli. The CNS Vital Signs program^1^ was used for all neuropsychological testing (Gulani and Sundgren, 2006).

### MRI data acquisition

For participants in the experimental group, MRI examinations were performed using a General Electric Discovery MR750 3.0-T instrument (General Electric Co. Ltd., CT) at Xijing Hospital of the Fourth Military Region. Standard T1-weighted, three-dimensional anatomical data were obtained using the Spoiled Gradient Recalled Echo sequence, with the following parameters: repetition time = 2,530 ms, echo time = 3.5 ms, flip angle = 7°, resolution matrix = 256 × 256, number of slices = 192, slice thickness = 1.0 mm, and voxel size = 1.0 × 1.0 × 1.0 mm^3^. fMRI data were acquired with an echo planar imaging sequence covering the entire brain, and the scanning parameters were as follows: repetition time = 2,000 ms, echo time = 30 ms, flip angle = 90° field of view = 220 × 220 mm^2^, acquisition matrix = 64 × 64, slice thickness = 4 mm, number of slices = 30, voxel size = 3.5 × 3.5 × 4.0 mm^3^, and number of volumes = 180. General Electric Discovery MR750 3.0-T scanners and the aforementioned consistent scanning parameters were used for the collection of all follow-up rs-fMRI and structural MRI data in the Lhasa at Xizang Military General Hospital. Both scanners received routine standardized periodic calibration during the research period to guarantee stable scanning performance.

For participants in the control group, MRI examinations had been performed using a 3.0-T Siemens Trio MRI scanner (Siemens Medical, Erlangen, Germany) at the Southwest University Center for Brain Imaging. For the collection of high-resolution T1-weighted anatomical images, a magnetization-prepared rapid gradient echo sequence was applied, and the scanning parameters were as follows: repetition time = 1,900 ms, echo time = 2.52 ms, inversion time = 900 ms, flip angle = 9°, resolution matrix = 256 × 256, number of slices = 176, slice thickness = 1.0 mm, and voxel size = 1.0 × 1.0 × 1.0 mm^3^. For the acquisition of fMRI data, the echo planar imaging sequence was applied,, and the scanning parameters were as follows: repetition time = 2,000 ms, echo time = 30 ms, flip angle = 90°, field of view = 220 × 220 mm^2^, slice thickness = 3 mm, slice gap = 1 mm, number of slices = 30, and voxel size = 3.4 × 3.4 × 3 mm^3^.

### rs-fMRI data preprocessing

Preprocessing of rs-fMRI data was carried out using the Resting-State fMRI Data Analysis Toolkit Plus (RESTplus).^2^ The following procedures were applied: format conversion from Digital Imaging and Communications in Medicine (DICOM) to Neuroimaging Informatics Technology Initiative (NIfTI); removal of the first 10 points; slice timing; realignment to correct for head motion (excessive motion defined as translation or rotation > 2 mm or 2°, respectively); spatial normalization to the Montreal Neurological Institute (MNI) space via resampling of 3 × 3 × 3 mm^3^ volumes; spatial smoothing using a 6-mm full-width at half maximum (FWHM) Gaussian kernel; removal of linear detrending; regression for nuisance covariates; and band-pass filtering at 0.01–0.08 Hz.

### Static and dynamic functional network construction

The static functional connectivity (sFC) network was constructed using GRETNA (Wang et al., 2015). sFC was calculated using the 116-region of interest (ROI) atlas with a radius of 3 mm. Specifically, 116 anatomically defined regions were mapped among all cerebral and cerebellar gray matter based on the Automated Anatomical Labeling (AAL) atlas (Tzourio-Mazoyer et al., 2002). We then extracted the mean time series for each region and calculated Pearson’s correlation coefficients. This analysis generated a 116 × 116 sFC matrix for each participant. The data were converted to *z*-values, which followed a near normal distribution, via Fisher’s z transformation. The rs-fMRI signal value for each node was calculated by averaging the preprocessed signals for all voxels within each ROI. The FC strengths were represented as edges between all possible pairs of nodes, which were defined by Fisher’s z-transformed Pearson correlation coefficients between the rs-fMRI time series. To better explain the results, each of the 116 AAL ROIs was further assigned to functional networks, including the visual network (VN), sensorimotor network (SMN), dorsal attention network (DAN), frontoparietal network (FPN), ventral attention network (VAN), limbic system (LS), default mode network (DMN), and basal ganglia (BG) (Hu et al., 2022; Schaefer et al., 2018).

The dynamic FC (dFC) network was constructed via the sliding-time window approach (Allen et al., 2014) using the AAL-116 atlas as the ROI template through the DynamicBC toolbox, which captures pairwise correlations of ROI time series within predefined time windows (Liao et al., 2014). Window length was set to 50 repetition times (100 s), with a sliding step of 2 repetition times. After exclusion of the initial 10 volumes during preprocessing, 170 valid time points remained. With this parameter setup, a total of 61 independent dFC matrices were generated to depict the dynamic fluctuation of functional connectivity across scanning.

### Graph theory analysis of sFC and dFC networks

The GRETNA toolbox (version 2.0.0)^3^ was used to calculate the graph-theoretical properties of both sFC networks and dFC networks. Sparsity thresholds ranging from 0.05 to 0.40 with an incremental step of 0.01 were applied for both sFC and dFC networks (Allen et al., 2014; Tang et al., 2024). This range avoids severely fragmented networks at extremely low sparsity and artifact-rich over-dense networks at high sparsity, ensuring biologically plausible topological quantification. At the global network level, we calculated the clustering coefficient (Cp), local efficiency (Eloc), characteristic path length (Lp), global efficiency (Eglob), and small-world properties gamma (γ), lambda (λ), and sigma (σ). At the nodal level, we computed degree centrality (Dc), betweenness centrality (Bc), nodal efficiency (Ne), nodal local efficiency (NLe), and nodal clustering coefficient (NCp). For all graph metrics, the area under the curve (AUC) across the sparsity range was computed to provide a threshold-independent characterization of network topology. Brain network hubs were defined based on nodal-level metrics (Dc, Ne, NLe, and NCp). Consistent with standard connectomics practice (Power et al., 2013), a node was classified as a hub if its AUC value for a given metric exceeded the network mean by at least one standard deviation. Nodes meeting this criterion were considered functionally central to interregional communication.

### Statistical analysis

Demographic and neuropsychological data were analyzed using SPSS 29.0 software (SPSS, Inc.). Subjective ratings of memory and reaction time were compared using one-way repeated-measures analysis of variance (ANOVA) at a specific time level (Time 1, Time 2, and Time 3), and *post-hoc* Bonferroni’s test was applied with a within-subject factor of condition in the experimental group. Partial eta-squared (ηp^2^) values were calculated to reflect effect size.

One-way repeated-measures ANOVA was separately applied to identify significant differences in the global and regional sFC and dFC network properties in the experimental and control groups at three time points, and correction was achieved using a false discovery rate (FDR) of *Q* = 0.05. For the ANOVA, we used MATLAB (R2023a) to extract the average time series for significantly different brain regions and nodes within each case and evaluated the changes at each time level (Time 1, Time 2, and Time 3). To gain insights into potential differences, paired *t*-tests comparing data from all pairs of the three time points (Time 1 vs. Time 2, Time 2 vs. Time 3, and Time 1 vs. Time 3) were performed for the experimental and control groups. All *p*-values from these paired *t-*tests were corrected for multiple comparisons using the FDR procedure at a threshold of *Q* = 0.05. Notably, the primary findings of this study were derived from a within-group design (longitudinal changes under HA exposure). Data from the control database were only used as supplementary evidence to verify whether age influences brain imaging results, rather than for a core between-group comparison.

For any measure showing cognition-related alterations, Pearson correlation analysis was applied to detect correlations between changes in brain functional indices and changes in neuropsychological features. Correlations were considered significant at a threshold of FDR-corrected *p*<0.05 (*Q* = 0.05) to control for Type I error.

## Results

### Establishment of the HA immigrant cohort

The present study enrolled young adult students who immigrated to a HA region of Tibet to pursue a college education. We screened a total of 69 healthy right-handed high school graduates of the Shaanxi–Tibet immigrant cohort (mean age, 18.624 ± 0.989 years; 37.68% female). Twenty of these students were excluded from the present study due to incomplete MRI data or questionnaire responses. The final experimental group included 49 students with a mean age of 18.734 ± 0.839 years and a female proportion of 34.69%. The average cumulative HA exposure time from Time 1 to Time 2 was 560.8 ± 7.3 days (range: 541–573 days), and the average cumulative exposure time from Time 2 to Time 3 was 552.4 ± 7.5 days (range: 535–568 days). A full description of this cohort was published with our previous study (Li et al., 2026).

In the control group, the average number of days between the first and second scanning sessions was 515 days, and that between the second and third scanning sessions was 520 days. The control group also included 49 healthy college students (19.31 ± 0.71 years, 34.69% female) from the SLIM Brain Data Repository.

### HA exposure induced impairments in working memory and psychomotor function

In this 4-year longitudinal study, we systematically assessed the effects of HA exposure on cognitive and neurobehavioral functioning by administering standardized neuropsychological tests to participants in the experimental group at baseline and at 2- and 4-year follow-up evaluations. The results demonstrated that HA exposure decreased accuracy on the verbal/visual memory test and prolonged response times on the visual/auditory reaction time test. The detailed results for cognitive function testing in this cohort have been described previously (Li et al., 2026; Supplementary Table 1).

### HA exposure led to alterations in static Dc and Ne in some brain regions

Building upon our prior findings concerning FC (Zeng et al., 2026), the present study extended the analysis to topological properties and explored the underlying mechanisms of HA-induced alterations in cognitive function. First, based on static topological properties, we analyzed changes in nodal network properties.

**FIGURE 1:**
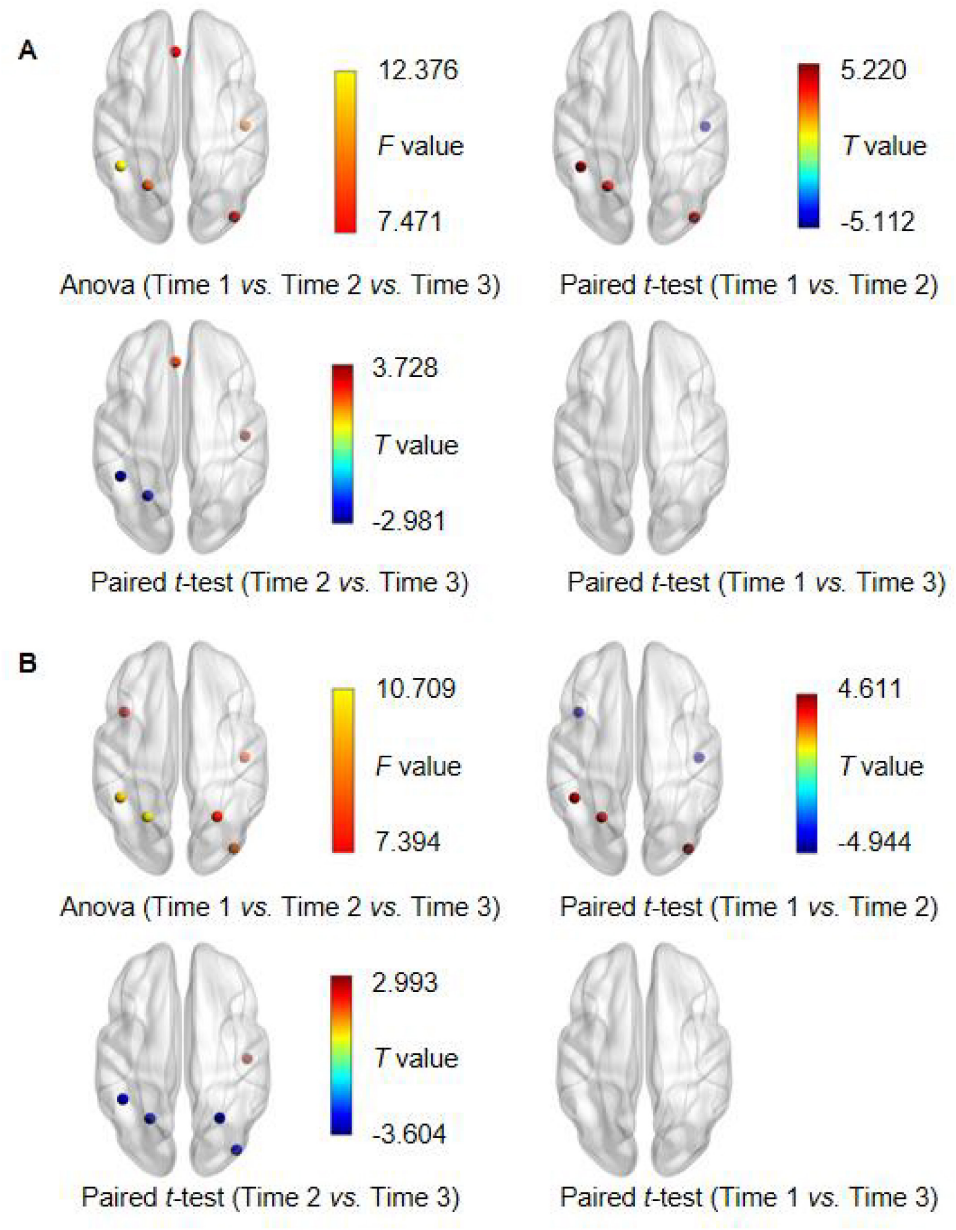
Changes in static degree centrality and nodal efficiency were observed in some regions after HA exposure. **(A)** Degree centrality. **(B)** Nodal efficiency.

In the experimental group, significant differences in Dc and Ne values were observed within 5 and 6 brain regions, predominantly within VN, DAN, FPN, and SMN (all uncorrected *p* < 0.001; Figure 1 and Table 1). Notably, the right inferior occipital gyrus (IOG.R), left superior parietal gyrus (SPG.L), left inferior parietal lobule (IPL.L), and right heschl gyrus (HES.R) exhibited concurrent changes in both Dc and Ne. *Post-hoc* multiple comparisons further demonstrated that within the SPG.L, IPL.L, and HES.R, Dc and Ne were significantly suppressed at Time 2 but had fully recovered by Time 3, with no significant differences detected between Time 3 and Time 1 (all FDR corrected *p* < 0.05). In contrast, no significant main effects were observed for any static regional network property in the control group (all uncorrected *p* > 0.001).

**TABLE 1:** Hub regions of FC networks differ after HA exposure (*n* = 49).

Graph metric	One-way repeated measures ANOVA ^a^			Paired *t*-tests ^b^ (Time 1 vs. Time 2)			Paired *t*-tests ^b^ (Time 2 vs. Time 3)			Paired *t-*tests ^b^ (Time 1 *vs.* Time 3)		
	Nodes	*F*	*p*	Nodes	*t*	*p*	Nodes	*t*	*p*	Nodes	*t*	*p*
Dc	ACG.L	7.536	0.001	IOG.R	4.403	<0.001	ACG.L	2.500	0.016	−	−	−
	IOG.R	7.471	0.001	SPG.L	4.080	<0.001	SPG.L	−2.611	0.012	−	−	−
	SPG.L	9.080	<0.001	IPL.L	5.220	<0.001	IPL.L	−2.981	0.004	−	−	−
	IPL.L	12.376	<0.001	HES.R	−5.112	<0.001	HES.R	3.728	0.001	−	−	−
	HES.R	9.611	<0.001	−	−	−	−	−	−	−	−	−
Ne	IOG.R	8.686	<0.001	IOG.R	4.611	<0.001	IOG.R	−2.947	0.005	−	−	−
	SPG.L	10.709	<0.001	SPG.L	4.116	<0.001	SPG.L	−3.408	0.001	−	−	−
	SPG.R	7.900	0.001	IPL.L	4.296	<0.001	SPG.R	−3.604	0.001	−	−	−
	IPL.L	9.901	<0.001	HES.R	−4.944	<0.001	IPL.L	−3.317	0.002	−	−	−
	HES.R	8.269	<0.001	TPOsup.L	−4.348	<0.001	HES.R	2.993	0.004	−	−	−
	TPOsup.L	7.394	0.001	−	−	−	−	−	−	−	−	−

^a^Uncorrected *p* < 0.001 for one-way repeated measures ANOVA.

^b^FDR corrected *p* < 0.05 for paired *t-*tests. ACG.L, left anterior cingulate and paracingulate gyri; Dc, degree centrality; FC, functional connectivity; HA, high altitude; HES.L, left heschl gyrus; IOG.R, right inferior occipital gyrus; IPL.L, left inferior parietal, but supramarginal and angular gyri; Ne, nodal efficiency; SPG.L, left superior parietal gyrus; SPG.R, right superior parietal gyrus; TPOsup.L, left temporal pole: superior temporal gyrus.

Notably, despite the significant changes in Dc and Ne for the SPG.L, IPL.L, and HES.R over the 4-year HA exposure period, these alterations were not associated with cognitive impairments (all *p* > 0.05).

### Static clustering coefficient and lambda increased after HA exposure

**FIGURE 2:**
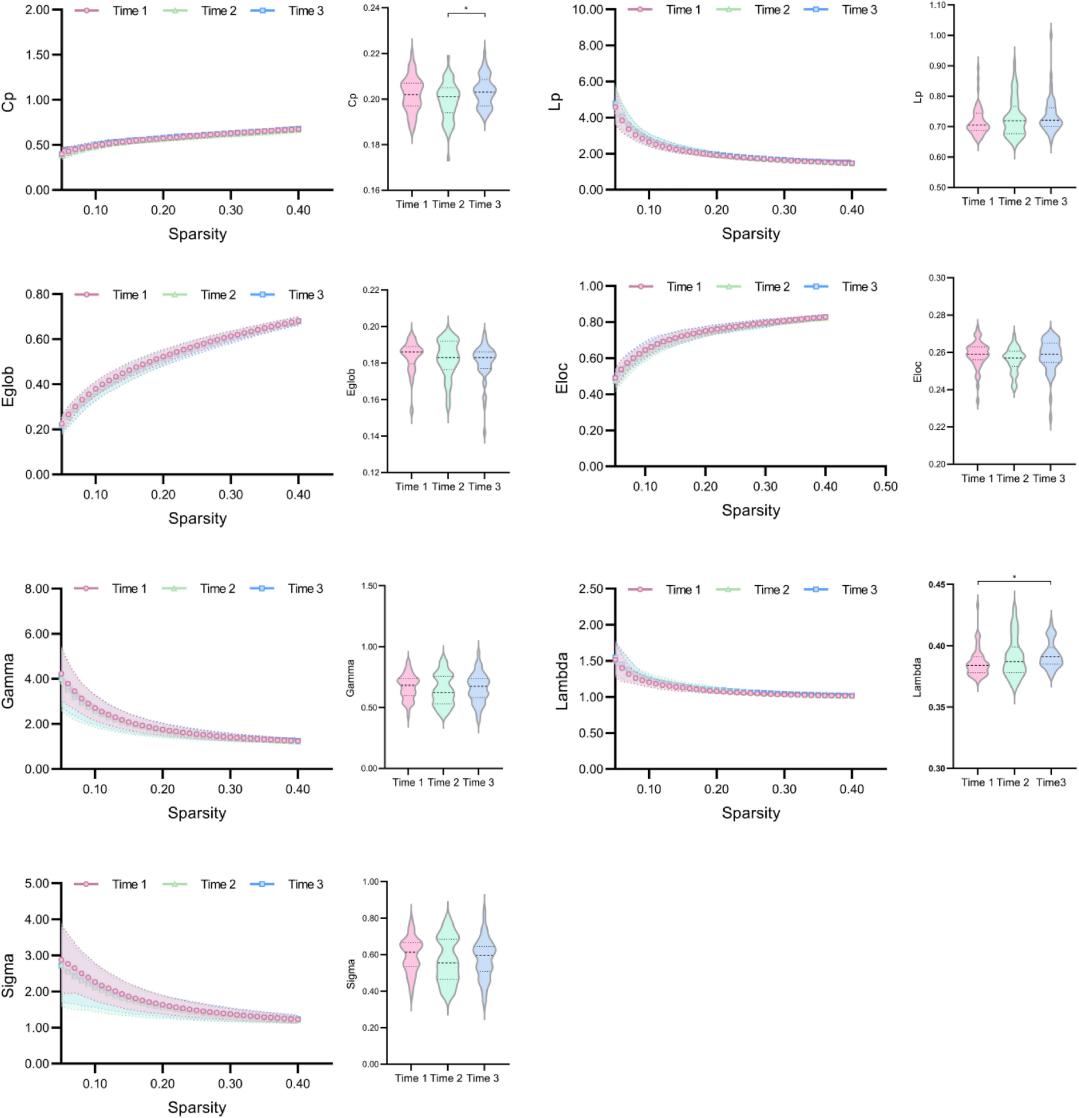
Static clustering coefficient and lambda increased after HA exposure. The left column represents the network efficiency and small-world properties characterized by γ > 1, λ≈1, and σ > 1 across the sparsity range of 0.05–0.40. The right bar demonstrates the group effect based on AUC values. The horizontal lines above the bars indicate comparisons that achieved statistically significant thresholds (*p < 0.05, Bonferroni adjusted). Cp, clustering coefficient; gamma, normalized clustering coefficient; HA, high altitude; lambda, normalized characteristic path length; Lp, characteristic path length; sigma, small-worldness index; Eglob, global network efficiency; Eloc, local network efficiency.

To further explore brain functional alterations at the global level induced by chronic HA exposure, we examined changes in global network properties over the 4-year follow-up period. The static functional connectomes generated for Time 1, Time 2, and Time 3 all exhibited small-world topologies, displaying high gamma (γ > 1) and near-unity lambda (λ≈1) values, which could be represented by the scalar σ (σ > 1) (Bassett and Bullmore, 2006; Figure 2).

In the experimental group, significant differences in the AUC values for Cp and lambda were observed across the three time points (all *p* < 0.05). *Post-hoc* multiple comparisons further indicated that the AUC for Cp was significantly increased only for the comparison of Time 3 with Time 2 (*p* < 0.05), with no significant difference detected between Time 2 and Time 1 (*p* > 0.05; Figure 2 and Supplementary Table 2). Furthermore, the AUC for lambda showed a significant increase only for the comparison of Time 3 to Time 1 (*p* < 0.05). However, the alterations in lambda did not exert a significant influence on changes in sigma. Comparatively, no significant main effects were observed for any static global network properties in the control group (all *p* > 0.05); Supplementary Figure 1 and Supplementary Table 2).

Although the AUC values for Cp and lambda showed alterations during the 4-year HA exposure period, neither property exhibited a statistically significant correlation with cognitive impairment (all *p* > 0.05).

### Biphasic alterations of dynamic nodal properties with chronic HA exposure

Compared to traditional sFC approaches, which assess nodal and global network properties under the assumption of temporal stationarity (i.e., functional connections remain stable over the observation period), dFC can offer insight into time-varying network organization within the brain. To expand upon the results of dFC analysis, we further investigated alterations in graph theory from a dynamic perspective.

**FIGURE 3:**
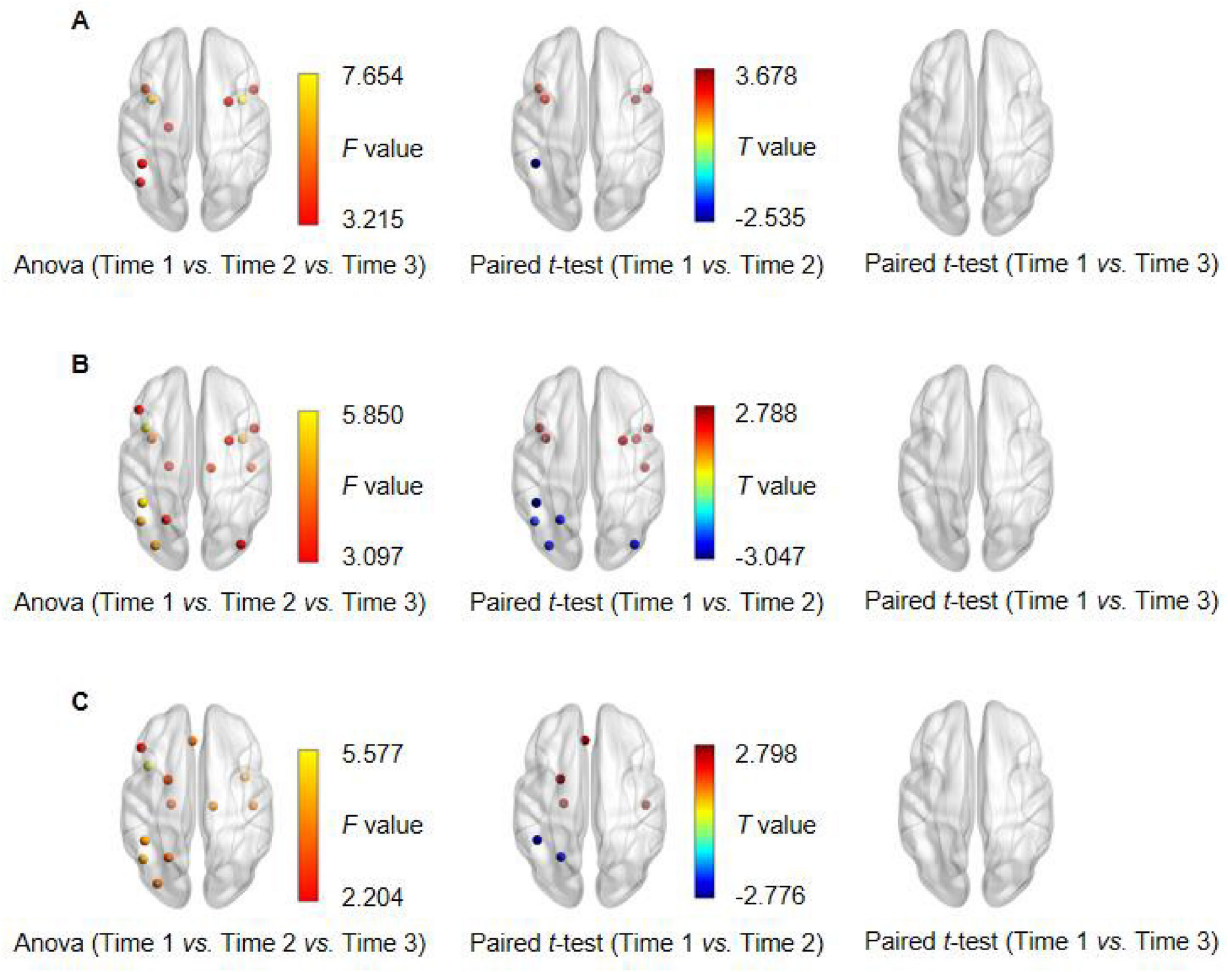
Dynamic nodal network properties exhibited a biphasic pattern characterized by initial suppression followed by full recovery during the 4-year HA exposure period. **(A)** Ne; **(B)** NLe; and **(C)** NCp. HA, high altitude; NCp, nodal clustering coefficient; Ne, nodal efficiency; NLe, nodal local efficiency.

We first examined alterations in dynamic nodal network properties over the 4-year HA exposure period. In the experimental group, Ne, NLe, and NCp varied significantly within 8, 14, and 12 brain regions, respectively. The predominant regions were within networks like the VN, DAN, FPN, SMN, VAN, LS, DMN, and BG (all uncorrected *p* < 0.001; Figure 3 and Table 2). Notably, the INS.L and PUT.R displayed concurrent alterations in both Ne and NLe; the INS.R, PHG.L, IPL.L, ANG.L, and TPOsup.L demonstrated concurrent alterations across Ne, NLe, and NCp; and the HES.R, MOG.L, SPG.L, and THA.R displayed concurrent alterations in both NLe and NCp (all uncorrected *p* < 0.001). *Post-hoc* multiple comparisons further demonstrated that within the INS.L, INS.R, IPL.L, TPOsup.L and TPOsup.R, the variances of Ne and NLe were only significantly suppressed at Time 2 (all FDR corrected *p* < 0.05). Meanwhile, within the SPG.L, IPL.L, and HES.R, the variances of NLe and NCp exhibited significant suppression only in the comparison of Time 2 and Time 1 (all FDR corrected *p* < 0.05). *Post-hoc* multiple comparisons further demonstrated that within the INS.L, INS.R, IPL.L, TPOsup.L and TPOsup.R, the variances of Ne and NLe were significantly suppressed only at Time 2. Meanwhile, consistent with the observed static nodal network properties, within the SPG.L, IPL.L, and HES.R, the variances of NLe and NCp exhibited significant suppression only in the comparison between Time 2 and Time 1. Overall, no significant differences were detected between Time 3 and Time 1. Moreover, these analyses revealed no associations between alterations in network properties and changes in cognitive performance (*p* > 0.05).

**TABLE 2:** Hub regions of dFC networks differ after HA exposure (*n* = 49).

Graph metric	One-way repeated measures ANOVA ^a^			Paired *t*-tests ^b^ (Time 1 vs. Time 2)			Paired *t-*tests ^b^ (Time 2 vs. Time 3)			Paired *t-*tests ^b^ (Time 1 vs. Time 3)		
	Nodes	*F*	*p*	Nodes	*t*	*p*	Nodes	*t*	*p*	Nodes	*t*	*p*
Ne	INS.L	6.506	0.002	INS.L	2.783	0.008	−	−	−	−	−	−
	INS.R	7.654	0.001	INS.R	3.678	0.001	−	−	−	−	−	−
	PHG.L	3.215	0.043	IPL.L	−2.535	0.015	−	−	−	−	−	−
	IPL.L	3.714	0.027	TPOsup.L	2.456	0.018	−	−	−	−	−	−
	ANG.L	3.224	0.043	TPOsup.R	2.847	0.007	−	−	−	−	−	−
	PUT.R	3.376	0.037	−	−	−	−	−	−	−	−	−
	TPOsup.L	4.089	0.019	−	−	−	−	−	−	−	−	−
	TPOsup.R	3.822	0.024	−	−	−	−	−	−	−	−	−
NLe	IFGtriang.L	3.122	0.047	INS.L	2.788	0.008	−	−	−	−	−	−
	INS.L	4.274	0.016	INS.R	2.469	0.017	−	−	−	−	−	−
	INS.R	4.854	0.009	MOG.L	−2.416	0.020	−	−	−	−	−	−
	PHG.L	3.414	0.036	MOG.R	−2.372	0.022	−	−	−	−	−	−
	MOG.L	4.843	0.009	SPG.L	−2.460	0.018	−	−	−	−	−	−
	MOG.R	3.097	0.048	IPL.L	−3.047	0.004	−	−	−	−	−	−
	SPG.L	3.355	0.038	ANG.L	−2.163	0.036	−	−	−	−	−	−
	IPL.L	5.415	0.005	PUT.R	2.282	0.027	−	−	−	−	−	−
	ANG.L	4.819	0.009	HES.R	2.618	0.012	−	−	−	−	−	−
	PUT.R	3.296	0.040	TPOsup.L	2.405	0.020	−	−	−	−	−	−
	THA.R	3.679	0.028	TPOsup.R	2.460	0.018	−	−	−	−	−	−
	HES.R	3.895	0.023	−	−	−	−	−	−	−	−	−
	TPOsup.L	5.850	0.004	−	−	−	−	−	−	−	−	−
	TPOsup.R	3.197	0.044	−	−	−	−	−	−	−	−	−
NCp	IFGtriang.L	3.204	0.044	ACG.L	2.732	0.009	−	−	−	−	−	−
	INS.R	4.229	0.016	PHG.L	2.677	0.010	−	−	−	−	−	−
	ACG.L	3.611	0.030	SPG.L	−2.552	0.014	−	−	−	−	−	−
	PHG.L	3.241	0.042	IPL.L	−2.776	0.008	−	−	−	−	−	−
	MOG.L	3.620	0.029	PUT.L	2.671	0.010	−	−	−	−	−	−
	SPG.L	3.593	0.030	HES.R	2.796	0.007	−	−	−	−	−	−
	IPL.L	4.434	0.014	−	−	−	−	−	−	−	−	−
	ANG.L	4.617	0.011	−	−	−	−	−	−	−	−	−
	PUT.L	3.292	0.040	−	−	−	−	−	−	−	−	−
	THA.R	4.027	0.020	−	−	−	−	−	−	−	−	−
	HES.R	3.771	0.025	−	−	−	−	−	−	−	−	−
	TPOsup.L	5.577	0.005	−	−	−	−	−	−	−	−	−

^a^Uncorrected *p* < 0.001 for one-way repeated measures ANOVA.

^b^ FDR corrected, *p* < 0.05 for paired *t-*tests. ACG.L, left anterior cingulate and paracingulate gyri; ANG.L, left amygdala; Dc, degree centrality; dFC, dynamic functional connectivity; HA, high altitude; HES.R, right heschl gyrus; IFGtriang.L, left inferior frontal gyrus, triangular part; INS.L, left insula; INS.R, right insula; IPL.L, left inferior parietal, but supramarginal and angular gyri; MOG.L, left middle occipital gyrus; MOG.R, right middle occipital gyrus; Ne, nodal efficiency;PHG.L, left parahippocampal gyrus; PUT.R, right lenticular nucleus, putamen; SPG.L, left superior parietal gyrus; THA.R, right thalamus; TPOsup.L, left temporal pole: superior temporal gyrus; TPOsup.R, right temporal pole: superior temporal gyrus.

In the control group, despite significant differences in the variance of Bc and Dc within 8 and 5 brain regions, respectively, *post-hoc* multiple comparisons did not identify consistent alterations in corresponding brain regions across the different time points (all uncorrected *p* < 0.001; Supplementary Table 3).

### Variance of Lp and Eglob increased with chronic HA exposure and correlated significantly with cognitive impairments

**FIGURE 4:**
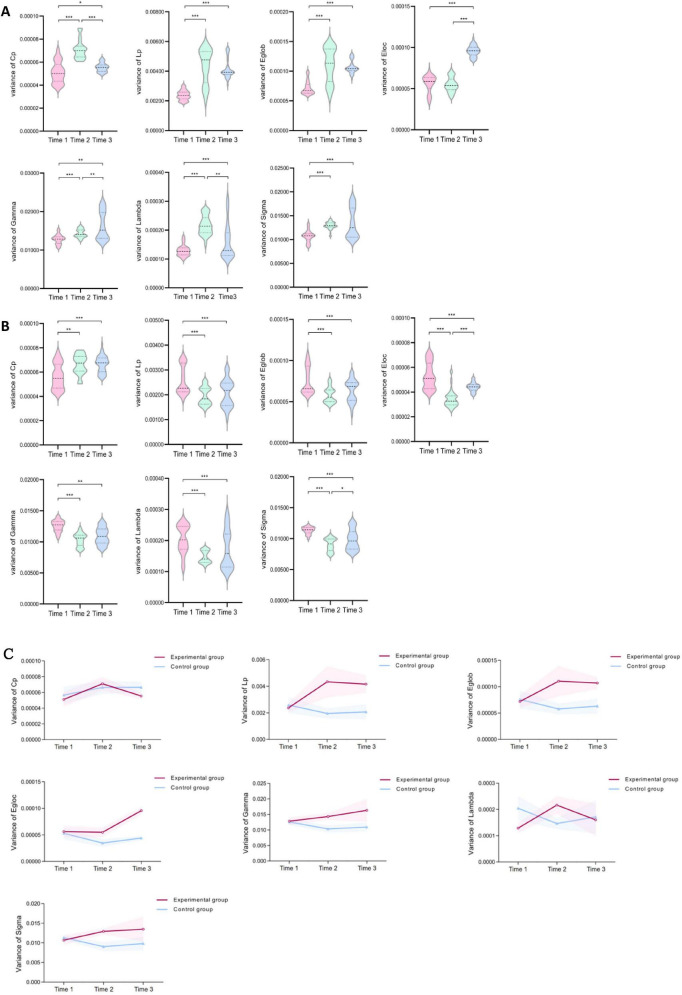
The variability of global network properties in dynamic functional connectivity were altered with HA exposure. The bar demonstrates the group effect based on AUC values. The horizontal lines above the bars indicate comparisons that achieved statistically significant thresholds (*p < 0.05, ^**^p < 0.01, ^***^p < 0.001, Bonferroni adjusted). Variance of global network properties: **(A)** in the experimental group; **(B)** in the control group; and **(C)** comparison of experimental group and control group.

To further investigate HA exposure-induced brain functional alterations at the global level, we examined changes in dynamic global network properties during the 4-year study period. Relative to matched random networks, the dynamic functional connectomes across three time points all showed small-world topologies. In the experimental group, the variances of Cp, Lp, Eglob, Egloc, gamma, lambda, and sigma differed significantly across the three time points (all *p* < 0.001, Figure 4 and Table 3). *Post-hoc* analyses further clarified the specific patterns of these differences: for Cp and lambda, variances were significantly increased at Time 2 versus Time 1, significantly decreased at Time 3 versus Time 2, and still significantly increased at Time 3 versus Time 1 (all *p* < 0.05); the variances of Lp, Eglob, and sigma all exhibited a consistent upward trend, being significantly increased at both Time 2 and Time 3 compared with Time 1 (all *p* < 0.001); and for Egloc, variance was significantly increased at Time 3 compared with both Time 1 and Time 2 (all *p* < 0.001). Regarding the small-world topologies, the variance of gamma was significantly increased across all three time points (all *p* < 0.01). Overall, all measured properties exhibited elevated values relative to baseline after 4 years of HA exposure.

**TABLE 3:** Graph theory changes in dynamic functional networks.

Graph theoretical metric								
Group	Network parameter	Time 1 (mean±SD)	Time 2 (mean±SD)	Time 3 (mean±SD)	*F*	*p*	η_p_^2^	*Post-hoc* comparison
Experimental group (*n*=49)	Variance of Cp	0.000051±0.000001	0.000071±0.000001	0.000055±0.000001	117.836	<0.001	0.711	Time 1<time 2 (*p*<0.001) Time 1<Time 3 (*p*=0.012) Time 2>Time 3 (*p*<0.001)
	Variance of Lp	0.002377±0.000047	0.004339±0.000166	0.004159±0.000093	112.474	<0.001	0.701	Time 1<Time 2 (*p*<0.001) Time 1<Time 3 (*p*<0.001)
	Variance of Eglob	0.000072±0.000002	0.000111±0.000004	0.000107±0.000002	58.277	<0.001	0.548	Time 1<Time 2 (*p*<0.001) Time 1<Time 3 (*p*<0.001)
	Variance of Eloc	0.000056±0.000001	0.000055±0.000001	0.000096±0.000001	299.156	<0.001	0.862	Time 1<Time 3 (*p*<0.001) Time 2<Time 3 (*p*<0.001)
	Variance of gamma	0.012828±0.000176	0.014302±0.000154	0.016336±0.000520	21.803	<0.001	0.312	Time 1<Time 2 (*p*<0.001) Time 1<Time 3 (*p*=0.001) Time 2<Time 3 (*p*=0.001)
	Variance of lambda	0.000129±0.000003	0.000216±0.000005	0.000160±0.000009	66.193	<0.001	0.580	Time 1<Time 2 (*p*<0.001) Time 1<Time 3 (*p*<0.001) Time 2>Time 3 (*p*=0.005)
	Variance of sigma	0.010694±0.000170	0.012943±0.000108	0.013483±0.000448	21.110	<0.001	0.305	Time 1<Time 2 (*p*<0.001) Time 1<Time 3 (*p*<0.001)
Control group (*n*=49)	Variance of Cp	0.000057±0.000001	0.000066±0.000001	0.000067±0.000001	18.868	<0.001	0.282	Time 1<Time 2 (*p*=0.001) Time 1<Time 3 (*p*<0.001)
	Variance of Lp	0.002593±0.000081	0.001956±0.000051	0.002071±0.000079	26.918	<0.001	0.359	Time 1>Time 2 (*p*<0.001) Time 1>Time 3 (*p*<0.001)
	Variance of Eglob	0.000076±0.000002	0.000058±0.000001	0.000063±0.000002	26.698	<0.001	0.374	Time 1>Time 2 (*p*<0.001) Time 1>Time 3 (*p*<0.001)
	Variance of Eloc	0.000053±0.000002	0.000035±0.000001	0.000044±0.000001	127.582	<0.001	0.727	Time 1>Time 3 (*p*<0.001) Time 2<Time 3 (*p*<0.001) Time 1>Time 3 (*p*<0.001)
	Variance of gamma	0.012563±0.000131	0.010343±0.000149	0.010933±0.000193	60.214	<0.001	0.556	Time 1>Time 2 (*p*<0.001) Time 1>Time 3 (*p*=0.001)
	Variance of lambda	0.000205±0.000006	0.000146±0.000003	0.000172±0.000009	25.466	<0.001	0.347	Time 1>Time 2 (*p*<0.001) Time 1>Time 3 (*p*<0.001)
	Variance of sigma	0.011324±0.000082	0.009028±0.000147	0.009818±0.000236	49.096	<0.001	0.506	Time 1>Time 2 (*p*<0.001) Time 2<Time 3 (*p*=0.033) Time 1>Time 3 (*p*<0.001)

Cp, clustering coefficient; Lp, characteristic path length; Eloc, local efficiency; Eglob, global efficiency.

In the control group, the variances of Cp, Lp, Eglob, Egloc, gamma, lambda, and sigma also differed significantly across the three time points (all *p* < 0.05; Figure 4 and Table 3). The variance of Cp was significantly increased at Time 2 versus Time 1, significantly decreased at Time 3 versus Time 2, and still significantly increased at Time 3 versus Time 1 (all *p* < 0.01). The variances of Lp, Eglob, Egloc, lambda, gamma, and sigma followed the same trajectory, being significantly lower at Time 3 versus Time 1 (all *p* < 0.05).

Interestingly, the variance trajectories for dynamic global network properties in the experimental group were markedly distinct from those observed in the control group. Specifically, the variances in experimental group gradually increased and stabilized at a high level, whereas those of control group remained lower throughout the study period.

**FIGURE 5:**
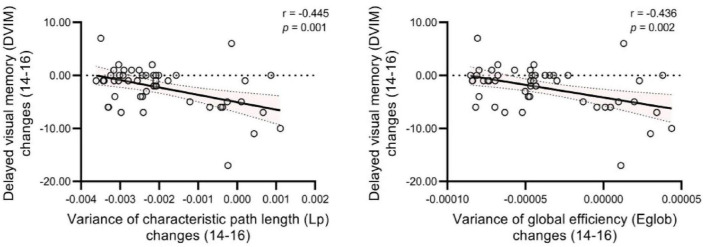
Correlations between variance of graph theory changes and cognitive impairments in the experimental group. Both variance of Lp and Eglob changes were negatively correlated with the accuracy changes of DVIM after 2 years HA exposure. *p* < 0.01, FDR-corrected. DVIM, delayed visual memory; Eglob, global network efficiency; HA, high altitude; Lp, characteristic path length.

Correlation analyses revealed significant relationships between changes in dynamic global network properties and cognitive impairments in the experimental group. Specifically, the variances of Lp and Eglob were negatively correlated with changes in DVIM (r _variance_ _of_ _Lp_ = −0.445, *p*_variance_ _of_ _Lp_ < 0.01; r _variance_ _of_ _Eglob_ = −0.436, *p*_variance_ _of_ _Eglob_ < 0.01; Figure 5).

## Discussion

HA-induced cognitive impairment represents a significant unresolved public health issue in HA regions. Existing research has largely centered on characterizing the adverse neural consequences of acute HA exposure (Chen et al., 2019; Chen et al., 2017; Zhang et al., 2022). Our earlier investigations, in which we integrated FC analyses (both static and dynamic) with behavioral assessments, revealed that FC perturbations are critical mediators of hypoxia-induced cognitive decline. Given that graph topological properties offer analytical advantages beyond FC for characterizing complex systems, the present study included a rigorous analysis of topological properties to provide further insight regarding the mechanisms of hypoxia-induced cognitive changes. The identified topological alterations may serve as a mechanistic basis for HA-induced cognitive impairment.

In this study, several nodal metrics exhibited significant alterations after 4 years of HA exposure. Notably, within the SPG.L, IPL.L, and HES.R, both Dc and Ne exhibited an initial suppression followed by a full recovery during the 4-year study period. This temporal pattern is consistent with observations from a previous study (Xin et al., 2020). The SPG and IPL are core components of the parietal lobe and are primarily responsible for spatial cognition, attention regulation, somatosensory integration, and multisensory information processing (Lin et al., 2022; Numssen et al., 2021). In contrast, the HES, which is located in the superior temporal plane, contributes to early auditory signal processing, prosody perception, attentional modulation, and social-emotional cognitive functions (Xue et al., 2022). In the early stages of HA exposure, the hypoxic environment inherent to HA settings may impose transient physiological stress on the brain. Specifically, within the parietal regions SPG.L and IPL.L, hypoxia could temporarily impair neuronal energy metabolism, thereby weakening the capacity of the tissues there to sustain dense local neural connections (as reflected by reduced Dc) and efficient global information transmission (as indicated by reduced Ne). Such functional perturbations may manifest as subtle decrements in the aforementioned cognitive functions during the early HA adaptation period. Furthermore, hypoxia might transiently disrupt the excitability of auditory cortical neurons within the HES.R, leading to diminished efficiency in local auditory signal integration and compromised coordination between the auditory cortex and other functionally associated brain regions. Based on existing evidence regarding chronic HA acclimatization, we speculate that two key biological mechanisms underpin this adaptive recovery: activity-dependent neuroplasticity and optimized cerebral metabolic function. Chronic hypoxic stimulation can drive synaptic remodeling, circuit reorganization, and enhanced neuronal connectivity to repair hypoxia-related functional deficits (Zhou et al., 2021). Meanwhile, adaptive metabolic regulation, including elevated hypoxia-inducible factor expression, improved oxygen utilization efficiency, and adjusted cellular energy metabolism, helps maintain stable neuronal activity in low-oxygen environments (Aboouf et al., 2023; Gong et al., 2025). Collectively, these plastic and metabolic adaptations restore the functional integrity of SPG.L, IPL.L, and HES.R, and sustain normal spatial cognition, attention, and auditory functions with long-term HA exposure.

The small-world network of the human brain is capable of both efficient separation processing and extensively integrated information processing (Suo et al., 2017). In this study, static small-world properties were consistent in the functional networks of the experimental and control groups, but only the experimental group exhibited significant changes in Cp and lambda with HA exposure. Specifically, Cp values increased significantly by the later stage of HA exposure (2–4 years), whereas the significant increase in lambda was observed only after 4 years of HA exposure. The Cp is a key graph-theoretical metric that quantifies local connectivity density and reflects the specialized processing efficiency of brain networks (Li J. et al., 2024), and notably, in this study, Cp did not exhibit sustained long-term changes despite its intermediate-phase fluctuation. The absence of a significant Cp difference between Time 3 and Time 1 confirmed that any temporary Cp elevation during the 2–4 year period represented a short-lived adaptive response to intermediate HA hypoxia (e.g., transient strengthening of local synaptic couplings to address metabolic stress) rather than a lasting modification induced by the HA environment. By the 4-year time point, Cp values had reverted to baseline levels, ultimately demonstrating that the HA environment did not induce sustained changes in the brain network’s local connectivity as measured by Cp. To evaluate alterations in small-world properties, we focused on lambda, gamma, and sigma. We observed a significant increase in lambda only between Time 3 and Time 1. Notably, this change in lambda did not exert a significant influence on changes in sigma; despite the isolated elevation of lambda at Time 3, sigma remained unchanged at a statistically significant level, demonstrating that the HA environment did not disrupt or alter the core small-world properties of the brain network. Given that sigma directly represents the core small-world structure, its stability provides strong evidence that the HA environment does not alter the brain network’s capacity to maintain small-world properties. Taken together, these results indicate that chronic HA exposure is not able to cause topological property alterations from the static global perspective.

Moreover, the shared pattern of suppression and recovery for both static and dynamic properties in the SPG, IPL, and HES reveals a unified neurocognitive adaptation trajectory to chronic HA exposure, one that simultaneously preserves stable cognitive function (via static properties) and enables flexible adjustment (via dynamic properties). In the SPG (attention), static Dc/Ne suppression at the 2-year time point showed disruption of the stable integration of the DAN, while the dynamic NLe/NCp suppression reflects impairment of the network’s flexible reconfiguration (e.g., shifting attention to new cues). Together, these deficits explain the observations of both sustained attention lapses (stable function) and poor adaptive focus (flexible function) after 2 years of HA exposure; later recovery of both property types ensures the DAN can reliably sustain attention and dynamically adjust to environmental changes. In the IPL (executive function), the static Dc/Ne losses weaken the FPN’s stable structural core, while the dynamic NLe/NCp losses undermine its flexible functional reconfiguration. This dual impairment leads to both working memory deficits (stable function) and cognitive performance (flexible function) after 2 years of HA exposure, and their combined recovery indicates restoration of the FPN’s ability to maintain reliable executive control and adapt to changing cognitive demands. In the HES (sensorimotor function), the static Dc/Ne reductions weaken the SMN’s stable sensorimotor links, while the dynamic NLe/NCp reductions impair its capacity for real-time adjustment (e.g., modifying motor responses to feedback). This dual disruption causes both reduced sensorimotor precision (stable function) and slow motor adaptation (flexible function) within 2 years, and recovery of both properties ensures the SMN can support consistent motor control and responsive adjustment to HA exposure. Collectively, these findings highlight that the brain’s adaptation to HA-related hypoxia is not limited to isolated changes in sFC or dFC, but rather a coordinated restoration of both property types, which ensures resilience across both stable and flexible domains of cognition.

Assessments of small-world properties in dynamic functional brain networks also revealed a propensity for abnormality, indicating some measurable impairments in brain networks with chronic HA exposure. Small-world properties are widely regarded as reflective of the balance of local neural specialization with global network integration. Abnormal small-world properties are observed in numerous diseases and are even considered to be causes of some diseases, including schizophrenia and Alzheimer’s disease (Mallio et al., 2015), in which alterations of network efficiency and clustering have been reported (Rubinov et al., 2009). Compared with baseline values, the variances of small-world properties (Cp, Lp, Eglob, Eloc, lambda, gamma, and sigma) in the dynamic brain networks exhibited a consistent increasing trend with prolonged HA exposure, suggesting a divergent and heterogeneous neuroplastic response likely driven by the synergistic effects of chronic hypobaric hypoxia and concomitant physiological stressors. Consistent with these results, previous studies observed the higher Eglob and Eloc variances in patients compared with healthy controls, indicating aberrant integration function of brain networks (Huang et al., 2022; Kim et al., 2017; Wang et al., 2020). Critically, a higher efficiency variance implies a higher degree of network efficiency fluctuation, ultimately reflecting a more unstable and inefficient condition. In the early stage of HA exposure, the brain enters an active adaptation stage in response to hypobaric hypoxia and physiological stressors. Neuronal connections (e.g., synaptic plasticity, functional coupling between brain regions) undergo extensive adjustments to adapt to the harsh environment. These large-scale, uneven adjustments directly lead to increased variances in small-world properties. After 2 years of adjustment, the functional network has formed a relatively balanced state that has adapted to the HA environment. Notably, variance had increased again at the 4-year follow-up, implying altered adaptive capacity in the late stage of chronic HA exposure. This phenomenon may result from the cumulative hypoxic burden and gradual exhaustion of compensatory mechanisms (Sarkar et al., 2003). Beyond potential oxidative stress induced by long-term hypoxia, enhanced neural noise or disrupted homeostatic regulation of network states under persistent metabolic stress could also account for the greater network fluctuations (Voytek and Knight, 2015). Specifically, excessive neural noise distorts regular neural signaling, while impaired homeostasis fails to maintain stable network activity, jointly driving unstable dynamic patterns across brain circuits. These complementary interpretations provide a more balanced view of the observed dynamic alterations.

In stark contrast, the low-altitude control group in this study exhibited a convergent trajectory with decreasing variance. This inverse pattern underscores that the HA environment serves as a potent neurobiological stressor, driving brain networks toward a state of elevated interindividual variability (Liu et al., 2024; Zhang and Zhang, 2022). In contrast, the low-altitude environment, characterized by stable normoxic conditions and minimal metabolic stress, supports uniform refinement of brain networks. Under such homeostatic conditions, interindividual differences in network metrics are attenuated, as neural systems converge toward a consistent functional or structural baseline. This contrast further validates the effect of environmental hypoxia on the variability of brain network organization, highlighting its capacity to unmask interindividual differences in neuroadaptive resilience.

Most importantly, our correlation analysis revealed a significant negative correlation between impaired cognition and altered variance of Lp and Eglob after 2 years of HA exposure. Distinct from reversible static nodal metrics, dynamic fluctuations in Lp and Eglob fail to recover, and such dynamic instability primarily accounts for sustained cognitive impairment. Specifically, increased Lp variance disrupts stable neural signal transmission required for time-sensitive cognition (e.g., sustained attention, sequential working memory), due to unpredictable interregional neural communication (Wee et al., 2014). Meanwhile, elevated Eglob variance fragments whole-brain global integration, undermining high-order cognitive processing (e.g., problem-solving, decision-making), which depends on coordinated cross-region information integration (Huang et al., 2022). In summary, persistent impairments in dynamic network reliability and integration, instead of recovered static nodal features, dominate chronic hypoxia-induced cognitive deficits. This indicates that static nodal recovery is functionally insufficient and highlights aberrant dynamic global network alterations as a key irreversible pathological biomarker of long-term hypoxic cognitive dysfunction.

The present study has several limitations that should be considered. First, the sample size was small, which could reduce statistical power, potentially obscuring significant effects and preventing the detection of subtle neurocognitive changes associated with chronic HA exposure. Future investigations in larger cohorts would enhance the reliability and generalizability of these findings. Second, the absence of longitudinal cognitive assessments in the control group represents a methodological constraint. Future longitudinal studies should incorporate matched sea-level control groups to better isolate the specific impacts of prolonged HA exposure. Third, the present study did not formally assess potential confounding factors including sleep quality and physical activity throughout the study period. These factors may jointly affect brain network characteristics and cognitive performance and should be collected and included as covariates in future relevant analyses. Differences in scanner systems between the HA and control samples constitute another limitation. Scanner-induced heterogeneity may alter signal quality and BOLD temporal properties, which could bias the calculation of static and dynamic FC. Such technical discrepancies further interfere with the reliable quantification of graph-theoretical topological metrics derived from connectivity matrices. Consequently, the between-group comparability of network topological properties may be compromised. Although we applied standardized preprocessing to reduce imaging artifacts, residual scanner-related variation cannot be fully eliminated. Future studies should ensure the use of uniform scanning equipment, or implement sensitivity analyses and harmonization techniques such as ComBat to calibrate cross-group datasets. These strategies will effectively reduce technical biases and strengthen the reliability of findings from graph theory analyses.

## Data Availability

The raw data supporting the conclusions of this article will be made available by the authors upon request.

## Footnotes

1
http://www.cnsvs.com/

2
http://www.restfmri.net/forum

3
https://www.nitrc.org/projects/gretna/
