# Supplementary material for: Alterations in static and dynamic topological properties of brain functional network after chronic high altitude exposure: a panel study
Source: Front Aging Neurosci. 2026 Jul 27;18:1773258. doi: 10.3389/fnagi.2026.1773258 (PMC13454118; doi:10.3389/fnagi.2026.1773258)
Supplement: Supplementary file 1 [file Supplementary_file_1.docx]

**Supplementary Table 1. Descriptive values and statistical analysis of the altered cognitive performance metrics after HA exposure (*n*=49)**

| Cognitive performance | Time 1  (Mean±SD) | Time 2  (Mean±SD) | Time 3  (Mean±SD) | *F* | *p* | η_p_^2^ | Post-hoc comparison |
| --- | --- | --- | --- | --- | --- | --- | --- |
| IVBM, n | 27.918±1.631 | 25.837±4.195 | 25.490±4.801 | 5.643 | 0.005 | 0.105 | Time 1>Time 2 (*p*=0.004)  Time 1>Time 3 (*p*=0.003) |
| IVIM, n | 23.122±2.579 | 22.367±2.885 | 20.878±3.492 | 8.249 | <0.001 | 0.147 | Time 1>Time 3 (*p*=0.003) |
| DVBM, n | 27.286±2.475 | 24.959±4.015 | 24.102±5.245 | 8.073 | 0.001 | 0.144 | Time 1>Time 2 (*p*=0.001)  Time 1>Time 3 (*p*=0.002) |
| DVIM, n | 21.592±2.992 | 20.061±3.602 | 19.469±3.680 | 7.677 | 0.001 | 0.138 | Time 1>Time 2 (*p*=0.012)  Time 1>Time 3 (*p*=0.003) |
| VSRT, s | 0.222± 0.113 | 0.352±0.090 | 0.399±0.084 | 62.939 | <0.001 | 0.567 | Time 1<Time 2 (*p*<0.001)  Time 1<Time 3 (*p*<0.001)  Time 2<Time 3 (*p*=0.012) |
| ASRT, s | 0.128±0.082 | 0.356±0.076 | 0.395±0.072 | 236.011 | <0.001 | 0.831 | Time 1<Time 2 (*p*<0.001)  Time 1<Time 3 (*p*<0.001)  Time 2<Time 3 (*p*=0.010) |
| VCRT, s | 0.399±0.128 | 0.524±0.179 | 0.609±0.179 | 22.237 | <0.001 | 0.317 | Time 1<Time 2 (*p*<0.001)  Time 1<Time 3 (*p*<0.001) |
| ACRT, s | 0.392±0.114 | 0.564±0.189 | 0.645±0.211 | 57.054 | <0.001 | 0.543 | Time 1<Time 2 (*p*<0.001)  Time 1<Time 3 (*p*<0.001)  Time 2<Time 3 (*p*=0.005) |

Note: n represents the number of correct responses on memory tests; s represents the seconds of reaction time; ACRT, auditory recognition reaction time; ASRT, auditory

simple reaction time; DVBM, delayed verbal memory; DVIM, delayed visual memory; HA, high altitude; IVBM, immediate verbal memory; IVIM, immediate

visual memory; VCRT, visual recognition reaction time; VSRT, visual simple reaction time.

**Supplementary Table 2. Graph theory changes in static functional networks**

|  |  | Time 1  (Mean±SD) | Time 2  (Mean±SD) | Time 3  (Mean±SD) | *F* | *p* | η_p_^2^ | Post-hoc comparison |
| --- | --- | --- | --- | --- | --- | --- | --- | --- |
| *Experimental group* (*n*=49) | Cp | 0.202±0.001 | 0.199±0.001 | 0.203±0.001 | 5.276 | 0.007 | 0.099 | Time 2<Time 3 (*p*=0.013) |
|  | Lp | 0.718±0.007 | 0.729±0.009 | 0.737±0.008 | 1.327 | 0.270 | 0.027 |  |
|  | Eglob | 0.183±0.001 | 0.182±0.001 | 0.181±0.001 | 1.144 | 0.323 | 0.023 | - |
|  | Eloc | 0.259±0.001 | 0.256±0.001 | 0.258±0.001 | 2.110 | 0.127 | 0.042 | - |
|  | Gamma | 0.670±0.015 | 0.643±0.018 | 0.659±0.017 | 0.598 | 0.552 | 0.012 | - |
|  | Lambda | 0.386±0.002 | 0.391±0.002 | 0.393±0.002 | 3.140 | 0.048 | 0.061 | Time 1<Time 3 (*p*=0.011) |
|  | Sigma | 0.386±0.002 | 0.569±0.017 | 0.578±0.016 | 0.725 | 0.487 | 0.015 | - |
| *Control group* (*n*=49) | Cp | 0.207±0.008 | 0.205±0.009 | 0.208±0.009 | 2.313 | 0.104 | 0.046 | - |
|  | Lp | 0.706±0.058 | 0.709±0.058 | 0.699±0.004 | 0.623 | 0.538 | 0.013 | - |
|  | Eglob | 0.185±0.009 | 0.185±0.011 | 0.187±0.007 | 0.489 | 0.615 | 0.010 | - |
|  | Eloc | 0.264±0.007 | 0.262±0.007 | 0.265±0.006 | 2.381 | 0.098 | 0.047 | - |
|  | Gamma | 0.693±0.114 | 0.706±0.115 | 0.695±0.106 | 0.197 | 0.821 | 0.004 | - |
|  | Lambda | 0.389±0.017 | 0.389±0.016 | 0.387±0.013 | 0.420 | 0.658 | 0.009 | - |
|  | Sigma | 0.612±0.107 | 0.623±0.114 | 0.615±0.100 | 0.169 | 0.845 | 0.004 | - |

Abbreviations: Cp, clustering coefficient; Lp, characteristic path length; Eloc, local efficiency; Eglob, global efficiency.

**Supplementary Table 3. Changes in hub regions of dynamic functional connectivity networks in the control group (*n*=49)**

|  | One-way repeated measures ANOVA ^a^ | | |  | Paired *t*-tests ^b^  (Time 1 *vs.* Time 2) | | |  | Paired *t*-tests ^b^  (Time 2 *vs.* Time 3) | | |  | Paired *t*-tests ^b^  (Time 1 *vs.* Time 3) | | |
| --- | --- | --- | --- | --- | --- | --- | --- | --- | --- | --- | --- | --- | --- | --- | --- |
|  | Nodes | *F* | *p* |  | Nodes | *t* | *p* |  | Nodes | *t* | *p* |  | Nodes | *t* | *p* |
| Bc | AMYG.R | 3.670 | 0.028 |  | AMYG.R | -2.859 | 0.006 |  | - | - | - |  | FFG.R | 3.336 | 0.002 |
|  | LING.L | 3.358 | 0.038 |  | IOG.L | -3.349 | 0.002 |  | - | - | - |  | ITG.R | 2.765 | 0.008 |
|  | IOG.L | 3.725 | 0.026 |  |  |  |  |  |  |  |  |  |  |  |  |
|  | FFG.L | 3.267 | 0.041 |  |  |  |  |  |  |  |  |  |  |  |  |
|  | FFG.R | 3.904 | 0.022 |  |  |  |  |  |  |  |  |  |  |  |  |
|  | PoCG.R | 3.875 | 0.023 |  |  |  |  |  |  |  |  |  |  |  |  |
|  | IPL.R | 3.330 | 0.039 |  |  |  |  |  |  |  |  |  |  |  |  |
|  | ITG.R | 3.246 | 0.042 |  |  |  |  |  |  |  |  |  |  |  |  |
| Dc | ACG.L | 3.314 | 0.039 |  | - | - | - |  | ACG.R | 2.669 | 0.010 |  | - | - | - |
|  | ACG.R | 4.158 | 0.018 |  | - | - | - |  | SOG.L | 2.798 | 0.007 |  | - | - | - |
|  | SOG.L | 3.648 | 0.028 |  |  |  |  |  |  |  |  |  |  |  |  |
|  | PoCG.L | 3.495 | 0.033 |  |  |  |  |  |  |  |  |  |  |  |  |
|  | PCL.R | 3.311 | 0.039 |  |  |  |  |  |  |  |  |  |  |  |  |

Note: ^a^ uncorrected *p<*0.001 for one-way repeated measures ANOVA; ^b^ FDR corrected *p* < 0.05 for paired *t*-tests. Abbreviation: Bc, betweenness centrality; Dc, degree centrality; ACG.L, left anterior cingulate and paracingulate gyri; ACG.R, right anterior cingulate and paracingulate gyri; AMYG.R, right amygdala; FFG.L, left fusiform gyrus; FFG.R, right fusiform gyrus; IOG.L, left inferior occipital gyrus; IPL.R, right inferior parietal, but supramarginal and angular gyri; ITG.R, right inferior temporal gyrus; LING.L, left lingual gyrus; PCL.R, right paracentral lobule; PoCG.L, left postcentral gyrus; PoCG.R, right postcentral gyrus; SOG.L, left superior occipital gyrus.

Supplementary Figure 1. Alterations of global topological properties in static functional connectivity in the control group. The left column represents the network efficiency and small-world properties across the sparsity range of 0.05–0.40. The right bar demonstrates the group effect based on AUC values. The horizontal lines above the bars indicate comparisons that achieved statistically significant thresholds (^*^*p*<0.05, Bonferroni adjusted). Abbreviations: Cp, clustering coefficient; gamma, normalized clustering coefficient; HA, high altitude; lambda, normalized characteristic path length; Lp, characteristic path length; sigma, small-worldness index; Eglob, global network efficiency; Eloc, local network efficiency.
